# Parvovirus B19: What Is the Relevance in Transfusion Medicine?

**DOI:** 10.3389/fmed.2018.00004

**Published:** 2018-02-01

**Authors:** David Juhl, Holger Hennig

**Affiliations:** ^1^Institute of Transfusion Medicine, University Hospital of Schleswig-Holstein, Lübeck, Germany

**Keywords:** parvovirus B19 infection, B19V, blood donors, transfusion-transmitted infection, blood donor screening

## Abstract

Parvovirus B19 (B19V) has been discovered in 1975. The association with a disease was unclear in the first time after the discovery of B19V, but meanwhile, the usually droplet transmitted B19V is known as the infectious agent of the “fifth disease,” a rather harmless children’s illness. But B19V infects erythrocyte progenitor cells and thus, acute B19V infection in patients with a high erythrocyte turnover may lead to a life-threatening aplastic crisis, and acutely infected pregnant women can transmit B19V to their unborn child, resulting in a hydrops fetalis and fetal death. However, in many adults, B19V infection goes unnoticed and thus many blood donors donate blood despite the infection. The B19V infection does not impair the blood cell counts in healthy blood donors, but after the acute infection with extremely high DNA concentrations exceeding 10^10^ IU B19V DNA/ml plasma is resolved, B19V DNA persists in the plasma of blood donors at low levels for several years. That way, many consecutive donations that contain B19V DNA can be taken from a single donor, but the majority of blood products from donors with detectable B19V DNA seem not to be infectious for the recipients from several reasons: first, many recipients had undergone a B19V infection in the past and have formed protective antibodies. Second, B19V DNA concentration in the blood product is often too low to infect the recipient. Third, after the acute infection, the presence of B19V DNA in the donor is accompanied by presumably neutralizing antibodies which are protective also for the recipient of his blood products. Thus, transfusion-transmitted (TT-) B19V infections are very rarely reported. Moreover, in most blood donors, B19V DNA concentration is below 1,000 IU/ml plasma, and no TT-B19V infections have been found by such low-viremic donations. Cutoff for an assay for B19V DNA blood donor screening should, therefore, be approximately 1,000 IU/ml plasma, if a general screening of blood donors for single donation blood components is considered at all: for the overwhelming majority of transfusion recipients, B19V infection is not relevant as well as for the blood donors. B19V DNA screening of vulnerable patients after transfusion seems to be a more reasonable approach than general blood donor screening.

## Introduction

Parvovirus B19 (B19V) has been described first in 1975: Cossard and colleagues (1) found “parvovirus-like particles” in the sera of nine blood donors and two patients. Meanwhile, these “parvovirus-like particles” are known to be B19V, a small, single-stranded DNA virus of approximately 5,500 nucleotides of which three different genotypes worldwide are existent. Genotype 1 is predominant worldwide, while genotype 2 is found only sporadically in Europe and the Americas. Genotype 3 seems to be widespread predominantly in north- and west-Africa (2). The diameter of B19V is between 19 and 25 nm, and it is a “bare” virus without any envelope. The icosahedral capsid consists of two structure proteins: VP1 and the smaller VP2 one. VP2 is the main protein in the capsid with a percentage of approximately 95% of the capsid while 5% of the capsid consists of VP1 (2, 3). The genome also encodes, besides for VP1 and VP2, for the non-structure protein NS1 which is essential for the replication of the viral DNA and responsible for the host cell apoptosis (4).

The tropism of B19V is very specific: B19V infects only cells with the blood group antigen P (globosid) on their surface (5). The antigen P is expressed on erythrocyte precursor cells and megakaryocytes as well as on endothelia cells, placental cells, and fetal myocardium.

Crucial for virus entry into the host cell is besides the P-antigen on the cell surface a distinct part of VP1, the unique amino-terminal region VP1u (6). The role of antibodies against epitopes on VP1 in the protection of erythrocyte progenitor cell and in the termination of the acute B19V infection underlines the importance of VP1 for the establishment of the infection by host cell entry (7, 8). Although B19V enters all cells with the antigen P, a productive infection with virus replication and the formation of progeny viruses happens only in erythrocyte progenitor cells, leading to the apoptosis of the infected cell.

However, many infections with B19V are asymptomatic or manifests only with mild of unspecific symptoms, flu-like symptoms, or arthralgia. The association of B19V with a disease was initially unclear (1) but only several years later, B19V could be linked to an exanthematous children’s disease, the erythema infectiosum or fifth disease (9).

The ability to enter P-antigen expressing tissues, may explain the distinct clinical picture of B19V infection in some patient at risk for a more severe course of the infection: acute infection causes an affection of a large number of erythrocyte precursors with consecutive apoptosis. Mass apoptosis of these cells may lead to a short-time arrest of hematopoiesis and slight drop of the hemoglobin value (10) or the hematocrit, respectively, but due to the life span of red cells of approximately 140 days, severe anemia due B19V infection in patients without high erythrocyte turnover are rare and only single cases have been reported (11–13). However, in patients with increased red blood cell destruction resulting in high erythrocyte turnover and a shorter half-life of red cells from other reasons, e.g., hemolytic anemia or hemoglobinopathies, already the short-time hematopoetic arrest due to B19V infection can cause a life-threatening aplastic crisis (4).

Penetration of other cells with the P-antigen on their surface can induce some other clinical pictures of B19V infection: the typical rash during the erythema infectiosum may be a sign of the B19V infection of cutaneous endothelial cells, although a deposit of immune complexes, formed by antigen-bound antibodies is also a possible explanation.

In non-immune, antibody-negative pregnant women, B19V-infection can be transmitted *via* the placenta to the unborn child. In first-trimester pregnancies, transplacental infection of the fetus can lead to miscarriage, in the second or third trimester, infection of fetal erythrocyte precursor cells in the liver, which is the site of fetal haematopoesis, and infection of myocardial cells causes a severe fetal illness with anemia and myocardial failure, a clinical picture that is called hydrops fetalis.

After the experimental infection of otherwise healthy volunteer subjects, the course of the B19V infection was characterized in detail first in 1985 (14): B19V is naturally droplet transmitted by aerosol *via* the upper respiratory tract, and the infection of the volunteers in this study was thus performed by intranasal inoculation with B19V. Already few days after infection, B19V was detectable in the plasma of the infected volunteers, and virus levels reached a peak 6–10 days after infection was induced. In this acute stage of infection, viral DNA at a concentration of more than 10^10^ IU/ml (15) plasma is detectable, followed by IgM antibody formation which precedes the appearance of IgG antibodies about some days (14, 16). Individuals, in whom IgG antibodies against B19V are present, are considered to be immune against a new infection with any B19V genotype.

Due to the infection path *via* droplet transmission, many infections occur during childhood: while in infants at an age of below 5 years, in only 2% antibodies against B19V are detectable as a marker of a past infection, the percentage of antibody-positive infants increases with the age: between an age of 5 and 9, in 21% of infants antibodies against B19V are detectable and 36% in adolescents between 10 and 19 years. In 49% of adults, between an age of 20 and 39 years, antibodies against B19V were detectable in this study (17). In Germany, in 66.9% of the adolescents at an age of 18–19 years, antibodies against B19V are detectable, also indicating that many infections occur already during childhood. Overall 72.1% of the adults between 18 and 79 years in Germany tested positive for anti-B19V-IgG as a marker for an infection anytime in the past (18).

## B19V Infection in Blood Donors

### Seroprevalence of B19V in Blood Donors

As shown by Anderson et al. (14) and known for other viral infections, also infection with B19V is accompanied by the formation of B19V-specific IgG antibodies, which are detectable for many years or even lifelong. The rate of B19V IgG-positive blood donors, the seroprevalence, thus serves for the assessment of the rate of donors who have had a B19V infection at any time in the past. Data about the prevalence of antibodies against B19V are available from several countries (Table 1). The seroprevalence differed between 9.78 and 79.1% in the different countries, but not only geographical differences might led to the differing seroprevalence rates but also differences in the numbers of investigated donors as well as different sensitivities in the antibody tests that were used.

**Table 1:** Seroprevalence, measured by anti-parvovirus B19 IgG determination, in blood donors.

Country	Seroprevalence (%)	No of investigated donors	Reference
Spain	9.78	92	(19)
India	27.96	1,633	(20)
Russia	29.70	1,000	(21)
Chile	55	400	(22)
Brazil	55.30	47	(23)
China	55.43	184	(24)
Brazil	60	100	(25)
Spain	64.70	136	(26)
Tunisia	65	378	(27)
Belgium	74	441	(27)
Italy	79.10	446	(28)

Data about the seroprevalence of B19V in German blood donors are lacking. However, the seroprevalence of B19V in blood donors is likely similar to that in the general adult population and can probably be transformed to the blood donor population: the seroprevalence of B19V in adults from the age of 18 years in Germany has been assessed to 72.1% and these individuals can be considered as probably immune against a B19V infection (18).

### Prevalence of B19V DNA in Blood Donors

Provided that 72% (or slightly less) of all blood donors are immune against B19V due to the presence of antibodies formed by a B19V infection in the past, the remaining blood donors without detectable antibodies are susceptible for a B19V infection. As the acute infection is often asymptomatic, especially in adults, affected blood donors are often not apparently ill and hence are allowed to donate blood. That way, viremic donations can be taken and B19V DNA can be detected in the plasma by nucleic acid testing (NAT). In the last years, with the availability of international standards (29, 30), it became common, to express the viral load of a blood sample by the quantity of B19V DNA in “International Units per milliliter (IU/ml)” rather than genome equivalents with one international unit being approximately equivalent to 0.6–0.8 genome equivalents (29).

Several reports about the prevalence of B19V DNA in blood donors or blood donations, respectively, have been published in the past 15 years. Investigation of blood donations for B19V DNA showed that detection of B19V DNA in blood donations is not a rare event: Already in studies performed in the 1990s, in 0.03–0.6% of all blood donations, detection of B19V DNA was reported (31–33). An overview about the prevalence of B19V DNA in blood donations as well as the number of investigated donations and the pool size is provided in Table 2.

**Table 2:** Prevalence of parvovirus B19 (B19V) DNA in blood donations.

Country	Prevalence of B19V DNA (%)	No of investigated donations	Pool size	Reference
UK	0.03	20,000	500	(31)
Japan	0.6	1,000	10	(32)
USA	0.1	9,568	50	(33)
Belgium	0.16	16,859	480	(34)
Portugal	0.12	5,025	10	(35)
Germany/Austria	0.013^a^ and 0.26^a^	2.8 million	96	(36)
USA	0.88	5,020	Single donation	(37)
The Netherlands	0.006	6.5 million	480	(38)
Korea	0.1	10,032	24	(39)
China	0.58	3,957	Single donation	(40)
Brazil	1	100	Single donation	(25)
Germany	0.61	53,789	96	(41)
China	0.06^b^	5,040	96	(42)
	0.079^b^	5,030	96	
Brazil	1	91	Single donation	(43)

*^a^Prevalence of B19V DNA was assessed for high (>10^6^ IU B19V DNA/ml plasma) and low (<10^6^ IU B19V DNA/ml plasma) viremic donations*.

*^b^Prevalence data were assessed for source plasma and for whole blood donors separately*.

The early prevalence data from the 1990s could be confirmed by more recently published reports: B19V DNA was present in one out of 625 (or 0.16%) donations in Belgium between 1999 and 2000 (34). As in many studies dealing with the issue of B19V DNA in blood donations, the screening for B19V DNA in this study was performed by minipool-testing: That means, not each single donation is tested for B19V DNA, but several donations are brought and tested together by NAT.

In another study from the USA, 5,020 archived samples collected in the years 2000 and 2003, were investigated for B19V DNA. The prevalence was 0.88% (37) and considerably higher compared to a further study that was performed in the Netherlands (38) between 2003 and 2009: 6.5 million blood donations have been tested for B19V DNA. However, donations with a concentration of B19V DNA below 1 × 10^6^ IU/ml plasma were not considered in the study. With the limitation that only donations with a high DNA concentration were considered, 411 out of the 6.5 million donations (or 0.006%) tested positive for B19V DNA.

In a Chinese study (40), 3,957 donor samples were screened for B19V DNA, 23 of those samples (0.58%) tested positive. In this study as well as in the study from the USA, only samples with lower DNA concentration had been detected (2.48 × 10^2^–6.38 × 10^4^ IU/ml and <20–1,869 IU/ml plasma), in contrast to the Dutch study. Acute B19V infections with high DNA concentrations seemed to be only shortly detectable, and thus, a large number of blood donations have to be screened for B19V DNA in order to detect a high viremic donation.

In another study from Austria and Germany (36), 2.8 million donations were screened for B19V DNA by minipools within 4 years. Minipools with a B19V DNA concentration >10^5^ IU/ml were resolved to identify the high viremic donation and the positive donation was discarded, but minipools with a lower B19V DNA concentration were not resolved. That way, 12.7 positive donations per 100,000 donations (0.13%) with a high viral load (>10^5^ IU/ml) were found and, presumably, 261.5 positive donations per 100,000 (0.26%) donations with a low (<10^5^ IU/ml) viral load.

In a recent study (41) concerning the issue of B19V DNA in blood donations was performed also in Germany: 53,789 donations from overall 23,889 donors (first time and repeat blood donors) were screened for B19V DNA within 1 year. In 326 donations (0.61%), B19V DNA was detectable, in most cases at a low concentration. Only in eight donations, more than 10^5^ IU B19V DNA/ml plasma were detectable.

Also the incidence as well as the prevalence of B19V DNA in blood donors were assessed in this study: 77/17,231 repeat blood donors were B19V DNA positive when tested first time during the study period and from 34/17,231 repeat blood donors, at least one negative sample were drawn, before they became infected during the study period. By these data, the prevalence of B19V DNA was calculated to be 0.45% and the annual incidence to be 0.20%.

The results of these studies are compatible with each other although a comparison of these studies is difficult due to differences in the size of the study population, the pool size [e.g., 480 donations in the Belgium study (34) vs 96 in the German/Austrian study (36) or vs single donation testing in the US study (37)] and use of different tests with a different analytical sensitivity for B19V DNA screening. Moreover, it is known that the occurrence of acute B19V infections follows a seasonal pattern, with many infections in spring and less or no infections in autumn (38). The point in time when a study was performed might, therefore, influence the prevalence of B19V DNA, especially if the study did not comprise the period of an entire year. It is also known, that in some years B19V epidemics occur, with a higher number of acute B19V infections in 1 year, while in other years only few B19V infections happen (38, 44).

## Course of the B19V Infection in Blood Donors

### Clinical Course

At present, there are no studies available which investigated systematically the clinical picture of the B19V infection in blood donors. However, in many otherwise healthy individuals like blood donors, the infection often goes unnoticed or presents with only mild or unspecific symptoms. One can assume that B19V infections in blood donors demonstrate a likewise course and from that reason, many B19V-infected blood donors appear to donate and are allowed to donate in the absence of any signs of disease.

### Hematological Course

As B19V affects erythrocyte precursor cells and megakaryocytes, a change in the hematological parameter (hemoglobin and platelets) could be expected in blood donors and was already described after the experimental infection of a low number of otherwise healthy individuals (14). The relation between B19V infection and blood count in blood donors has been investigated in one study in Germany (41): blood counts of 345 samples with detectable B19V DNA were compared to 100 B19V DNA-negative controls. While no differences in the quantity of leukocytes, erythrocytes, and platelets were observed, the mean hemoglobin value, the hematocrit, the mean corpuscular volume, the mean cellular hemoglobin value, and the mean hemoglobine concentration of the 345 B19V DNA-positive samples were statistical significant lower than the 100 controls. However, although statistical significant, the differences were only moderate and without any clinical relevance. Also, in the context of acute B19V infection with high DNA concentration, no major differences in the blood count could be observed in comparison to controls or to the B19V DNA-negative samples of the same donors which were drawn before the B19V infection.

### Course of the Viremia

The course of the viremia during B19V infection in blood donors is well investigated. The results of the first study, which investigated the course of the viremia after the infection, led to the conclusion that viremia is rapidly cleared in acutely infected, not immune compromised individuals (14). However, the method used to detect B19V DNA (dot-plot hybridization) was less sensitive compared to the methods that are currently used in the blood donor screening, but such a sensitive method was not available at that time. Especially in recent studies, the concentration of B19V DNA, measured in the plasma by NAT, serves as parameter for the level of the viremia of blood donors. And despite the use of more sensitive methods for the detection of B19V DNA, until the beginning of the last decade, the viremia in the acute B19V infection was considered as a rather short phenomenon, lasting only for several weeks (45, 46). The duration of viremia in blood donors has been estimated to be 17.5 days (95% CI 11.0–53.0) (47). The persistence of the virus or chronic infections were considered as extremely rare (48) and a longlasting viremia as a phenomenon that is limited to more severely ill and immunocompromised individuals rather than blood donors, although the data of Jordan and colleagues (33) already suggested that a longer period of viremia might be possible: at least one of the 11 B19V DNA-positive donors in this study was tested positive again during a follow-up investigation performed over 5 months later.

Compatible with this observation were the results of a longitudinal study from France (49): 76 patients who suffered from hemoglobinopathies, and were thus, like blood donors, not immunocompromised, have been followed up for several years. In six of these patients, persistence of B19V DNA at low levels (10–100 IU B19V DNA/ml plasma) for several years (up to 60 months) was determined. Also in a Japanese study, 20 B19V DNA-positive blood donors were followed up for several years and investigated for B19V DNA biannual. A decline of the B19V DNA concentration was found to be below 10^4^ IU B19V DNA/ml plasma after 1 year and 10^3^ IU/ml plasma after 2 years, subsequently, B19V DNA concentration persisted between 10^3^ and 10^1^ IU B19V DNA/ml plasma in the third and fourth year (50). In a German-Austrian Study (36), a rapid decline in the median B19V DNA concentration from 4.85 × 10^7^ to 4.6 × 10^2^ IU/ml within approximately 12 weeks was observed. Another study (51) investigated the duration of B19 viremia in 75 blood donors, in whom several consecutive samples could be investigated during 5.5 years. In this period, only in a minority of blood donors, the entire duration of viremia could be assessed, as the last B19V DNA-negative sample before infection *and* a B19V DNA-negative sample after the infection became available, indicating a long duration of viremia. However, the mean interval between drawing the first positive sample and the last positive samples in the study period was 21.5 months (range: 2.3–52.4 months; 95% CI: 19.1–23.9 months). Compatible with the rapid decline of B19V DNA concentration in the former studies (36, 41), a rapid decline of the B19V DNA concentration from a mean value of 2.23 × 10^8^ IU B19V DNA/ml plasma (95% CI: 0–6.48 × 10^8^ IU/ml plasma) to a mean value of 1,598 IU B19V DNA/ml plasma (95% CI: 1,157–2,039 IU/ml plasma) was assessed between the first B19V DNA-positive sample and the second sample, which has been taken after a mean time of 135.8 days later. B19V DNA then persisted in the donors who could be investigated for a longer period, for several years at low (10^2^–10^3^ IU/ml plasma) or very low levels (<10^2^ IU/ml plasma).

Persistence of B19V DNA has been proven (52–54) in several tissues (e.g., liver, heart, tonsils, synovia) and it has been suggested that, after acute infection, possibly bare B19V DNA, and *not* mature virions, are released from these tissues into the plasma (53, 55). A recent study provided evidence that the positive detection of B19V DNA in the plasma of blood donors approximately 6 months after acute infection is based on bare DNA strands and not on mature, infectious virions. These DNA strands may persist for years after the acute infection (56). Based on this assumption, most of the B19V DNA-positive donations, namely those with low DNA concentrations, might not be infectious for the recipients and the persistence of B19V DNA in blood donors after the decrease of the peak B19V DNA concentration might be irrelevant.

### Humoral Immune Response in B19V-Infected Donors

The humoral immune response in the B19V infection in blood donors has already been studied thoroughly and might influence the assessment of the importance in Transfusion Medicine. Already Anderson and colleagues (14) observed the typical antibody response in experimentally infected individuals with formation of anti-B19V IgM in the second week after infection, followed by the formation of anti-B19V IgG few days later. However, the epitope specificity (anti-NS1, anti-VP1, anti-VP2) of these antibodies was not reported.

Early studies on blood donors reported the prevalence of IgG and IgM antibodies in B19V DNA-positive blood donors: in the Netherlands (38), a subgroup of 67 out 411 B19V DNA-positive blood donors was investigated for antibodies of both classes. In 47 (70%) of the B19V DNA-positive donors, no antibodies against B19V were detected, neither of the IgM nor of the IgG class. 16 (24%) donors tested positive for anti-B19 IgM, and 4 (6%) tested positive for IgG *and* IgM. A further characterization of these antibodies was not performed and the findings of this study (no antibodies or predominant IgM detectable) are compatible with acute B19V infections in the investigated blood donors and not with longlasting infections. A German-Austrian study (36) was the first, which investigated the course of the humoral immune response in relation to the B19V DNA concentration by investigation of follow-up samples from 50 B19V-infected donors. IgG antibodies against epitopes on the viral capsids (VP1, VP2) were detectable in approximately one-third of the donors in the first sample, which has been taken during the acute infection. Already in the first follow-up sample, taken 12 weeks thereafter, in all of the donors, IgG antibodies against epitopes on the viral capsid, were detectable. These antibodies were also detectable in a second follow-up sample. As these IgG antibodies were directed against epitopes on the virus surface (VP1 and VP2), it can be hypothesized that these antibodies are able to neutralize the virions by hindering their binding to the cellular receptor on the target cells for B19V. This assumption was corroborated by another experiment in this study: samples with high B19V DNA concentrations and with low B19V DNA concentrations were filtered through a protein G column. Afterward, the DNA concentration was assessed again, and the reduction of B19V DNA after protein G filtration was significantly higher in samples with low DNA concentration compared to those samples with high DNA concentration. This finding indicated the presence of strong binding, high avide IgG antibodies on the surface of B19V, leading to clearance of B19V in the sample by adsorption of IgG-binded virions to the protein G *via* the Fc-fragment of the IgG.

In another study from Germany (51), the humoral immune response in 75 B19V-infected blood donors was investigated, 29 of them had an acute B19V infection during the study period, in the remaining donors, the infection occurred before the study period. Overall 410 samples with detectable B19V DNA have been provided by these donors within 5.5 years and could be considered in the study. That way, the course of B19V infection in blood donors could be studied over a longer period. Besides B19V DNA, samples were investigated for anti-B19V IgM, quantitative for anti-B19V IgG, and the avidity of anti-B19V IgG antibodies was determined. In only six samples with high B19V DNA concentrations, no anti-B19V IgG was detectable. The decrease of B19V DNA concentration was accompanied by an increase of the anti-B19V IgG titer, compatible with the findings of another study (41). Out of 29 donors with an acute, recently acquired B19V infection, in 24 (82.8%) already IgG antibodies, directed against epitopes on VP1 and VP2 with high avidity, were detectable. In five donors, no IgG antibodies against B19V were detectable in the first B19V DNA-positive donation, but at the point in time, when the next follow-up samples was drawn from these donors, also anti-B19V IgG with high avidity, directed against viral capsid proteins, were detectable. The study could also demonstrate that detection of anti-B19V IgM is not a suitable marker for the acute B19V infection as B19V IgM could not be detected in many donors during the acute infection.

## Transfusion-Transmitted (TT-) B19V Infection by Blood Products, in Particular by Single Donation Blood Products

Data suggesting a B19V transmission by plasma derivates are available since the 1990s (57, 58). Due to its physicochemical properties, B19V is hard to inactivate or to remove by processes used for other viruses during the manufacturing process of plasma derivatives. In addition, because of the prevalence of B19V DNA among blood donors, the entering of a donation with high DNA concentration in a plasma pool for fractionation is not a rare event, leading to the contamination of the plasma pool from several thousand donations. Thus, it is not astonishing that in a large number of plasma pools for fractionation, B19V DNA is detectable, when untested donations are entering in a pool (59) and that many TT-B19V infections by plasma derivatives have been reported in the past (57). To avoid further manufacturing of such plasma pools, B19V DNA testing of plasma pools or plasma units for fractionation is recommended by the Food and Drug Administration. The B19V DNA concentration should not exceed 10^4^ IU B19V DNA/ml plasma,^1^ a level that is considered as the maximum acceptable in plasma pools for fractionation. Nevertheless, there is further evidence for a B19V transmission by plasma derivatives despite the exclusion of plasma donations or plasma pools with high B19V DNA concentration for fractionation (60).

Measures to avoid potential B19V transmission by single donation blood products have not been established so far in many countries, although the first cases of TT-B19V infection were reported also in 1990s (61, 62). Despite the frequent detection of B19V DNA in blood donors, suspected TT-B19V infections are rarely reported in Germany, a country in which several millions of transfusions are carried out annually.^2^ If a case of viral transmission by transfusions is suspected in Germany, the German authority has to be informed according to the German transfusion act. However, the publications of this authority in which suspected cases of TT infection were reported during the period from 1997 to 2012, included no cases of suspected TT-B19V infection.^3^^,^^4^ In 2013, seven B19 infections in a donor and two in 2014 were reported, but infection of these donors never led to a transmission to the recipients of their blood components and no suspected cases of a TT-B19V infection due to an overt infection in a recipient of single-donor blood components was reported to the German authority by a treating physician.^5^ TT-B19V infections hence seem to be either a rare event or with a low clinical relevance, so that many infections are overlooked by the treating physicians. Also data reported to the Serious Hazard Of Transfusion (SHOT-) registry in the UK support this assumption: only one case of major morbidity due to a TT-B19V infection has been reported to SHOT between 1996 and 2016 (63).

Initially, only blood products provided from donors with high B19V DNA concentrations seemed to be infectious for their recipients. In a study from the USA, a fourfold increase in the IgG antibody titer in an already anti-IgG antibody-positive recipient of a red blood cell concentrate from a donor with 2.9 × 10^10^ IU B19V DNA/ml plasma was detected. The authors interpreted their finding as an anamnestic immune response, triggered by a TT-B19V infection through the red blood cell concentrate (15), but they detected no TT-B19V infection through blood products with a B19V DNA concentration of less than 10^6^ IU/ml plasma and concluded, that transmission with blood components from donors with a lower B19V DNA concentration do either not occur or are at least a rare event. Shortly later, a TT-B19V infection in a susceptible, antibody-negative recipient by a red blood cell concentrate from an antibody-negative donor with an acute B19V infection was reported. The B19V DNA concentration in the donor’s plasma was 5 × 10^9^ IU/ml, the genome of the B19V of the donor and the recipient shared 100% of their sequences, making the red blood cell concentrate as the origin of infection very probable (64).

However, the threshold DNA concentration in blood donors that was considered as being infectious for the recipients of their single donation blood products decreased more and more in the following years. Already in the year 2011, it could be demonstrated that red blood cell concentrates from B19V-infected donors with DNA concentration of 10^5^ IU/ml plasma are probably able to transmit B19V by their donation: after transfusion of red blood cell concentrates from nine out of 18 donors with a DNA concentration of 10^5^ IU B19V DNA/ml plasma or more, an infection in the recipients occurred, but no TT-B19V infection was observed after transfusion of red blood cell concentrates from donors with less than 10^5^ IU B19V DNA/ml plasma. In this study, phylogenetic analysis of B19V in the donors and the infected recipients yielded the blood products as the probable origin of infection (65). In donors with lower B19V DNA concentration (<10^5^ IU/ml plasma) without transmission of B19V infections to the recipients, a higher proportion of probably neutralizing antibodies was detectable. Besides the lower DNA concentration, the presence of such antibodies might also be protective for the recipients of the blood products. But already in the same years, transmissions of B19V infections through blood donations from donors with still lower DNA concentrations were reported: In a Japanese study (66), a TT-B19V infection through a red blood cell concentrate provided by a donor with a B19V DNA concentration of 5.1 × 10^3^ IU B19V DNA/ml plasma and proved by genome sequence analysis, occurred. Also IgG and IgM antibodies were detectable in the donor’s plasma, demonstrating that antibodies in the donor are not always protective for the recipient of his blood products. Although a further probable TT-B19V infection after transfusion of a red blood cell concentrate from a donor with low (10^3,2^ IU B19V DNA/ml plasma) DNA concentration and *with* detectable B19V IgG antibodies was reported (67), single donation blood products with such a low B19V DNA concentration, seem not to be infective in either case: no TT-B19V infections have been observed after transfusion of 15 single donation blood products (eight red blood cell concentrates, four pooled platelet concentrates, and three fresh frozen plasma) from donors with a B19V DNA concentration between 10^3^ and 10^4^ IU B19V DNA/ml plasma (68).

In the latest report of a TT-B19V infection, a red blood cell concentrate taken from a donor with a B19V DNA concentration of 1.1 × 10^4^ B19V DNA/ml plasma was the probable source of the infection. The B19V infection of the recipient has been accompanied by an immune thrombocytopenia (69).

It is noteworthy that, in only three of the recent studies, the symptomatology of the transfusion recipient finally led to the investigation of the donor: in one Japanese study (66), the infections in the recipients became evident due to more or less serious, miscellaneous symptoms like reticulocytopenia, delayed recovery of red blood cells after chemotherapy or pure red cell aplasia, and also rash or febrile disease. In France (67), a recipient suffered from erythroblastopenia after transfusion of a red blood cell concentrate due to sickle cell disease, and in Japan again, fever and thrombocytopenia led to the diagnosis of B19V infection (69). In the other studies (15, 64, 65, 68), positive screening of blood donors for B19V DNA induced the retrospective investigation of the recipients of their blood products, in whom no B19V infection had been suspected so far. Retrospective analysis of the red blood cell counts in one study (68) revealed a slight drop of the hemoglobin values in the two infected recipients after transfusion of red blood cell concentrate. However, the slight decrease of the hemoglobin level might also be attributed to other reasons like allogeneic blood stem cell transplantation, or iatrogen due to excessive blood sample withdrawal. No specific symptoms were specified later by the affected recipients in another study (66) and no symptoms in the recipients have been reported in two further studies (64, 65).

## Conclusion: What is the Relevance of B19V in Transfusion Medicine and What Can be Done?

Approximately 30% of the potential blood donors starting at an age of 18 years have no antibodies against B19V and are thus susceptible for new B19V infections. And as B19V infection often go unnoticed especially in adults, B19V-infected donors cannot be recognized by clinical symptoms or evident aberrances in the blood count and are, therefore, allowed to donate. From that reasons, detection of B19V DNA in blood donations is not a rare finding, at least in comparison to other transfusion-transmissible viral agents like HIV, HCV, and HBV. Unlike suggested in the past, B19 viremia after the acute infection is not a short phenomenon limited to several days or weeks, but persistence of B19V DNA for months to years, also in otherwise healthy blood donors, seems to be the norm rather than the exception and that way, multiple consecutive B19V DNA-positive donations can be taken even from a single donor. However, high DNA concentrations indicates peak viremia in the acute infection, but shortly after the acute infection DNA concentration decreases rapidly, accompanied by formation of potentially neutralizing IgG antibodies.

That TT-B19V infections are seldom reported by treating physicians may serve as an indication of the minor relevance of B19V for the transfusion of single donation blood components, either because they do not occur at all or because of their missing clinical consequences. In many reports, dealing with the issue of TT-B19V infection, only the detection of B19V DNA in the donor induced the investigation of the usually asymptomatic recipient of the blood products, and it was not a symptomatic transfusion recipient who was the cause for the investigation of a donor for a B19V infection. This could indicate that the overwhelming majority of TT-B19V infections (if they occur at all) are overlooked by the treating physicians either due to missing or only mild and unspecific symptoms and only single cases of TT-B19V infections with severe consequences were reported (66, 67, 69).

To avoid TT-B19V infections, several measures have been proposed: In Japan, screening for B19V is performed by hemagglutination assay to avoid donations with high viral load entering a plasma pool for fractionation (66, 70). However, the method is less sensitive, detects only donations with a very high viral load and, therefore, more suitable to detect plasma donation that should not enter a plasma pool for fractionation due to their high B19 viral load. As many single donations with lower viral loads and thus the potential to transmit B19V to recipient are overlooked by this assay, the performance in blood donor screening for single donation blood products is not reasonable.

The most efficient method to avoid TT-B19V infections is a general blood donor screening for B19V DNA by NAT. This can be also done by minipools, in which multiple donations are brought together for NAT screening. Screening assays nowadays have a sufficient sensitivity (36, 71, 72) to reliably detect donations even with minimal amounts of B19V DNA. On the basis of the current knowledge, maximal security in terms of avoiding a TT-B19V infection is provided, if the sensitivity of the assay is sufficient to detect a single donation with a B19V DNA concentration of 10^3^ IU B19V DNA/ml plasma.

In contrast to NAT screening of blood donations, anti-B19V IgM screening is not suitable to detect blood donations at risk for the transmission of a B19V infection: although IgM is generally regarded as formed during the acute B19V infection, IgM antibodies are not always detectable during the peak viremia in the acute infection stage (51). Reasons might be a pre-seroconversion acute infection when B19V DNA precedes the antibody formation or the disappearance of IgM antibodies during the Ig-class switch.

Another, antibody testing-based strategy for providing “B19V safe” single donation blood components has been proposed in the Netherlands (73): single-donor blood components can be considered as “B19V safe” if they are donated from donors, in whom anti-B19V IgG has been detected in two separate samples, taken after an interval of at least 6 months. And although B19V DNA is usually detectable over a longer period than 6 months after seroconversion (unlike believed at that time), the measured concentration is low, the detection of B19V DNA is probably based on bare DNA strands and, moreover, B19V DNA accompanied by the presence of protective antibodies in at least all donors with ongoing B19V infection. This approach warrants more protection against TT-B19V infection by single-donor blood products. However, anti-B19V IgG testing, preferably fully automated, is required just as an efficient algorithm, which warrants anti-B19V IgG testing after 6 months and then the declaration of single donation blood products as “B19V safe.”

Besides donor screening for B19V DNA, inactivation or removal by the virions is applied during the manufacturing process for plasma derivates. Measures like pasteurization or treatment with low pH were effective in the elimination of B19V (74) as well as nanofiltration (75) and an additional gain of security concerning the transmission B19V by plasma derivatives can be expected.

Some data are currently available concerning the effectiveness of pathogen reduction technologies like the amotosalen/UVA treatment or the riboflavin/UV-light treatment of cellular blood products in the inactivation of B19V. By such treatment, a considerably reduction of human or porcine B19V has been reported (76). However, a possible transmission of B19V by an amotosalen/UVA treated pooled platelet concentrate has been also reported recently (77), making a final conclusion about the effectiveness of pathogen inactivation of B19V in cellular blood products currently difficult.

Whether a general screening for B19V DNA or implementation of other measures for providing “B19V safe” single donation blood components is meaningful should be debated thoroughly. According to current knowledge, B19V seems to be of minor relevance in the administration of single-donor blood components. Symptomatic TT-B19V infection are very rarely reported by clinicians. This may be because they rarely occur for donor reasons: the viral load, if mature virions are present at all in the donor, is too low and/or circulating virions are neutralized by coexisting anti-B19V IgG antibodies with specificity for VP1, protecting the recipient against the infection. Also patient reasons are possible: the seroprevalence in the patient population is comparable to that of the general population. This means that approximately 70% of the transfusion recipients have antibodies against B19V and are probably immune. Another explanation is that TT-B19V infections occur, but are overlooked by the treating physician because they do not have any clinical relevance.

Unlike other transfusion-transmissible viruses (e.g., HBV, HCV, HIV), B19V infections can be well treated, e.g., by transfusion of further red blood cells or by administration of i.v. IgG, and thereby major consequences of TT-B19V infections can be prevented. Moreover, HBV, HCV, and HIV pose a threat for a majority or almost all transfusion recipients and cannot or hardly be community acquired, in contrast to B19V, which is droplet-transmissible but against which already 70% of transfusion recipients are immune.

Hence, another approach than a general blood donor screening to protect recipients for TT-B19V infection is to generate awareness in clinicians for the possibility of TT-B19V infections. Distinct susceptible patient groups for a more severe course of a potential B19V infection (e.g., pregnant women, immunosupressed patients, patients with high erythrocyte turnover) should be investigated thoroughly for symptoms of a B19V infection after transfusion and also performance of a B19V NAT screening in the patient can be considered. In this way, not only rarely occurring TT-B19V infections but also the presumably more frequently occurring community-acquired B19V infections can be detected.

## Author Contributions

DJ and HH reviewed the literature and wrote the manuscript.

## Conflict of Interest Statement

The authors declare that the research was conducted in the absence of any commercial or financial relationships that could be construed as a potential conflict of interest.

## Footnotes

^1^https://www.fda.gov/downloads/BiologicsBloodVaccines/GuidanceComplianceRegulatoryInformation/Guidances/Blood/ucm078510.pdf.

^2^http://www.pei.de/DE/infos/meldepflichtige/meldung-blutprodukte-21-transfusionsgesetz/berichte/berichte-21tfg-node.html.

^3^http://www.pei.de/SharedDocs/Downloads/vigilanz/haemovigilanz/publikationen/haemovigillanz-bericht-1997-2008.pdf;jsessionid=734FDB0D86C7B79C8E95E0E9AE86EF2F.1_cid329?__blob=publicationFile&v=1.

^4^http://www.pei.de/SharedDocs/Downloads/vigilanz/haemovigilanz/publikationen/haemovigilanz-bericht-2011.pdf?__blob=publicationFile&v=6.

^5^http://www.pei.de/SharedDocs/Downloads/vigilanz/haemovigilanz/publikationen/haemovigilanz-bericht-2013-2014.pdf?__blob=publicationFile&v=4.
